# Postsynaptic movement disorders: clinical phenotypes, genotypes, and disease mechanisms

**DOI:** 10.1007/s10545-018-0205-0

**Published:** 2018-06-13

**Authors:** Lucia Abela, Manju A. Kurian

**Affiliations:** 1 Molecular Neurosciences, Developmental Neuroscience, UCL Institute of Child Health, London, UK; 2 Developmental Neurosciences Programme, UCL GOS - Institute of Child Health, 30 Guilford Street, London, WC1N 1EH UK

## Abstract

Movement disorders comprise a group of heterogeneous diseases with often complex clinical phenotypes. Overlapping symptoms and a lack of diagnostic biomarkers may hamper making a definitive diagnosis. Next-generation sequencing techniques have substantially contributed to unraveling genetic etiologies underlying movement disorders and thereby improved diagnoses. Defects in dopaminergic signaling in postsynaptic striatal medium spiny neurons are emerging as a pathogenic mechanism in a number of newly identified hyperkinetic movement disorders. Several of the causative genes encode components of the cAMP pathway, a critical postsynaptic signaling pathway in medium spiny neurons. Here, we review the clinical presentation, genetic findings, and disease mechanisms that characterize these genetic postsynaptic movement disorders.

## Introduction

Movement disorders comprise a heterogeneous group of diseases characterized by either an excess of abnormal movements (hyperkinesia) or a lack of normal movements (hypokinesia) (Stoessl and Mckeown 2016). The phenotypes can be complex and overlapping, particularly in children, and can even change or evolve over time (Stoessl and Mckeown 2016; Kurian and Dale 2016). For many movement disorders, there are no biomarkers available to aid diagnosis. However, recent genetic advances have greatly contributed to improved diagnosis for patients with movement disorders (Olgiati et al. 2016; Reale et al. 2018). Over the past few years, a number of new genetic movement disorders have been identified, some of which are caused by alterations in genes involved in postsynaptic pathways. Indeed, defects in postsynaptic dopaminergic signaling in striatal medium spiny neurons are emerging as key drivers in the development of a number of genetic hyperkinetic movement disorders. In this review, we discuss the clinical presentation, management, genetic findings, and current understanding of contributory pathogenic mechanisms of such genetic movement disorders associated with striatal postsynaptic dysfunction.

### Synaptic physiology

Synapses are complex neuronal structures that are organized in several cellular compartments including the axon terminal membrane of the presynaptic neuron, the synaptic cleft, and the postsynaptic density (PSD) of the adjacent neuron. Synapses contain functionally and structurally distinct molecular machineries for synaptic connectivity and neurotransmission, the very essential processes that underlie brain function. Depending on the brain area, neurons interconnect with thousands of others and form dense, overlapping, and interdigitated networks that define the brain’s connectivity. Synaptic signaling is characterized not only by the anatomical organization of neurons but also by distinct neurotransmitter systems, which include amino acids (e.g., inhibitory GABA, excitatory glutamate), monoamines (e.g., dopamine, serotonin), peptides, purines, trace amines, and acetylcholine (Hyman 2005). In chemical synapses, arrival of electrical signal results in membrane depolarization and influx of calcium into the presynaptic terminal, which ultimately results in release of neurotransmitters into the synaptic cleft (Südhof 2013). Neurotransmission is a spatially and temporally precisely regulated process that involves the concerted interaction of specific proteins at the pre- and postsynaptic sites. Neurotransmitters are stored and transported in defined structures, known as synaptic vesicles (SVs). SVs are organized in distinct pools at the presynaptic terminal including a reserve pool, a recycling pool, and a primed or readily releasable pool (Rizzoli and Betz 2005). Release of the SV content involves a dedicated molecular machinery and includes several steps: SV priming, docking, and calcium-mediated fusion to the cell membrane (Rizo and Xu 2015). To ensure repetitive and sustained transmission, SVs have to be rapidly recycled. SV recycling is a complex process and involves several endocytic pathways for the retrieval of SV components from the plasma membrane and regeneration of functional SV (Kononenko and Haucke 2015; Soykan et al. 2016). Upon release, neurotransmitters diffuse across the synaptic cleft and bind to their respective receptors on the postsynaptic membrane which activate downstream signaling cascades. The receptors are attached to the postsynaptic density (PSD), which is a multi-protein complex organized into distinct layers of anchoring membrane molecules, scaffolding molecules, signaling molecules, and cytoskeleton molecules. The PSD is a specific feature of glutamatergic synapses. However, PSD-95, a key component of the PSD, has been also identified in glutamatergic synapses of midbrain dopaminergic neurons (Jang et al. 2015) and in medium spiny neurons of the human neostriatum (Morigaki and Goto 2015). The PSD is defined to receive and convert the chemical neurotransmitter signal into electrical and biochemical responses in the postsynaptic neuron (Sheng and Kim 2011). In general, the pre- and postsynaptic compartments are highly dynamic and modify their function or structure in response to specific synaptic activity.

### Synaptic pathology

Given the complex molecular organization of synapses, alterations of its composition, structure, or function can have a severe impact on neuronal function leading to neurological disorders (Waites and Garner 2011). Overall, synaptic dysfunction may occur at a number of different sites including the following: (1) the neuronal soma and axonal compartment affecting synaptic gene expression, SV synthesis, and trafficking; (2) the presynaptic compartment affecting SV exocytosis, endocytosis and recycling, maintenance of SV pools and proteostasis, and synaptic metabolic homeostasis involving mitochondrial function; (3) the intersynaptic compartment affecting neurotransmission and neurotransmitter recycling; and (4) the postsynaptic compartment affecting function of channels, receptors, and associated downstream signaling cascades. The association of human brain disorders with aberrant synaptic function and structure has led to the new concept of “human synaptopathy” (Lepeta et al. 2016). In recent years, synaptic dysfunction has been linked to a variety of neuropathological conditions including epilepsy (Hamdan et al. 2009; Caleo 2009; Casillas-Espinosa et al. 2012), movement disorders (Quartarone and Pisani 2011; Calo et al. 2016; Schirinzi et al. 2016; Calabresi et al. 2016; Matikainen-Ankney et al. 2016; Lepeta et al. 2016), intellectual disability (Mircsof et al. 2015; Crocker-Buque et al. 2016; Zapata et al. 2017; Ung et al. 2017), autism spectrum disorders (Giovedí et al. 2014; De Rubeis et al. 2014), psychiatric disorders (Kang et al. 2012; Fromer et al. 2014), and neurodegenerative disorders (Musardo and Marcello 2017). Recent advances in next-generation sequencing technologies and subsequent functional validation of identified genetic variants in patients with distinct neurological disorders have further contributed to understanding the genetic mechanisms underlying these human synaptopathies (Baker et al. 2015; Lipstein et al. 2017; Myers et al. 2017; Guarnieri et al. 2017; Sadybekov et al. 2017).

### Postsynaptic dysfunction in brain diseases

Recent isolation and proteomic profiling of the PSD of the human neocortex have revealed 1461 proteins (Bayés et al. 2011). Mutations in over 100 of these proteins cause brain diseases enriched in cognitive, affective, and motor phenotypes (Bayés et al. 2011). Over time, mutations have been identified in genes encoding postsynaptic receptors, ion channels, and components of associated signaling cascades, and the phenotypic spectrum is ever-expanding. Distinct populations of neurons often show a specific vulnerability to genetic alterations, depending on the genes, proteins, and neurotransmitters they express, and the neural circuits they are connected to. For example, GABAergic neurons are thought to play a key role in a number of genetic epilepsies. Mutations in GABA_A_ receptor subunits *GABRA*_*1*_, *GABRB*_*3*_, and *GABRG*_*2*_ have been identified in a broad spectrum of different epilepsy syndromes including Dravet syndrome, generalized seizures, epileptic encephalopathies, and febrile seizures (Johannesen et al. 2016; Shen et al. 2017; Niturad et al. 2017). Moreover, mutations in the *GRIN2A* gene encoding the NMDA glutamate receptor α2 subunit are emerging as a key genetic factor in the epilepsy-aphasia spectrum disorders (Kingwell 2013; Yang et al. 2017). Dysfunction of excitatory hippocampal neurons has been related to intellectual disability caused by mutations in genes encoding proteins of the PSD complex or interacting components (Zapata et al. 2017; Ung et al. 2017). Dysfunction of striatal medium spiny neurons (MSNs) due to alterations in genes encoding key postsynaptic proteins is associated with the pathogenesis of dystonia, dyskinesia, chorea, and parkinsonism.

### Key features of the dopaminergic postsynaptic medium spiny neuron

MSNs account for approximately 95% of all neurons in the striatum which represents the main input station of the basal ganglia, a group of distinct subcortical nuclei involved in motor control and behavior (Fisone et al. 2007). MSNs receive excitatory glutamatergic input from the cortex and the thalamus and modulatory dopaminergic input from the midbrain, in particular from the substantia nigra pars compacta, which innervates the dorsal-lateral striatum, and from the ventral tegmental area, which innervates the medial portion of the dorsal striatum and the ventral striatum (Fisone et al. 2007). Striatal MSNs give rise to inhibitory GABAergic projections to the globus pallidus (striatopallidal pathway) and the substantia nigra pars reticulata (striatonigral pathway).

#### Dopaminergic signaling in striatal medium spiny neurons

**Fig. 1:**
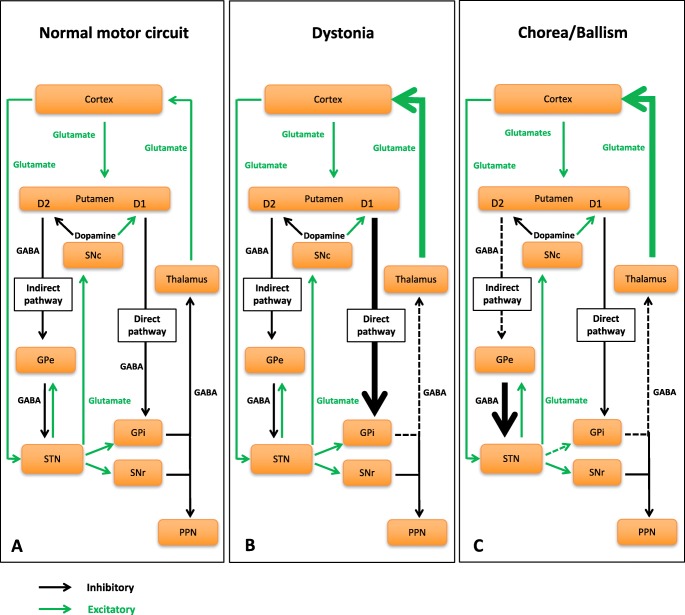
Basal ganglia motor circuits in normal physiology and hyperkinetic movement disorders. (a) Basal ganglia circuits in normal condition showing direct and indirect-pathway projections from dopaminergic neurons of the substantia nigra pars compacta to the subthalamic nucleus, the globus pallidus internal segment, and the substantia nigra pars reticulata. (b) Overactivation of the direct pathway in dystonia, and (c) hypofunction of the indirect pathway in chorea ultimately lead to disinhibition of thalamocortical neurons and hyperkinesia. SNc, substantia nigra pars compacta; Gpe, globus pallidus external segment; STN, subthalamic nucleus; Gpi, globus pallidus internal segment; SNr, substantia nigra pars reticulata; PPN, pedunculopontine nucleus (brainstem)

According to their output projections, neurotransmitters, and receptors, MSNs can be classified into two groups. D1-type dopamine receptor (DRD1)-expressing MSNs use enkephalin as a co-transmitter and project direct inhibitory monosynaptic fibers to the globus pallidus internal segment (GPi) and subthalamic nucleus (STN) (Chuhma et al. 2011). D2-type dopamine receptor (DRD2)-expressing MSNs use substance P as a co-transmitter and project indirect excitatory polysynaptic fibers to the same nuclei via the globus pallidus external segment (GPe) and STN (Chuhma et al. 2011) (Fig. 1(a)). It is generally believed that direct and indirect MSNs in the dorsal striatum exert opposite effects on the control of movement. Activation of DRD1 stimulates direct striatopallidal pathway MSNs and results in disinhibition of thalamocortical neurons, thus facilitating movement. Activation of DRD2, however, inhibits indirect striatonigral pathway MSNs and leads to inhibition of thalamocortical neurons and suppression of movement (DeLong et al. 2007). In clinical practice, hyper- and hypokinetic features often coexist, for example, in patients with parkinsonism-dystonia; the reasons for this are not entirely clear, but may be related to developmental age, indicating a complex disruption of basal ganglia motor circuitry.

#### cAMP signaling pathway in striatal medium spiny neurons

**Fig. 2:**
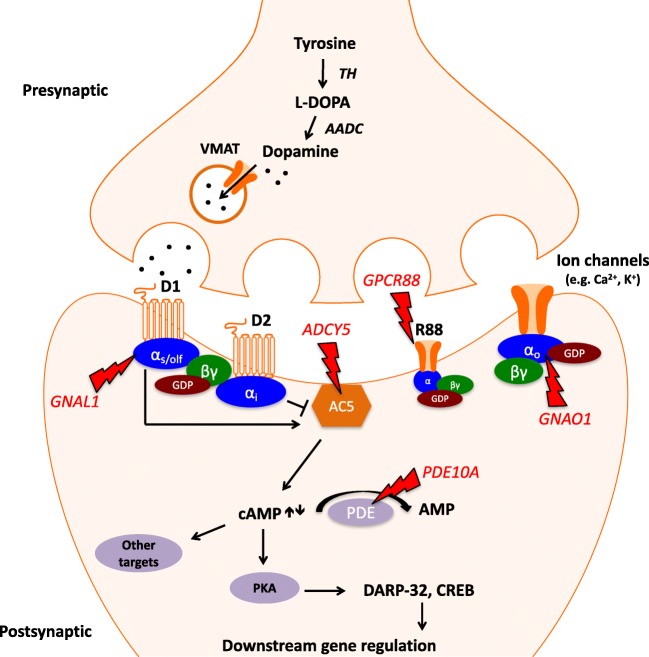
Schematic overview on a striatal medium spiny neuron synapse. Dopaminergic signaling in striatal medium spiny neurons is mediated by the cAMP signaling pathway. Activation of D1-type dopamine receptors leads to activation of adenylyl cyclase 5 and subsequent increase in cAMP levels, while activation of D2-type dopamine receptors results in inhibition of adenylyl cyclase 5 and reduced levels of cAMP. cAMP in turn modulates activity of the protein kinase A, which phosphorylates further downstream effectors including DARP-32 and CREB. Arrows indicate mutations in genes involved in postsynaptic dopaminergic signaling in striatal medium spiny neurons

Signaling through DRD1 and DRD2 in postsynaptic MSNs is mainly mediated by the G-protein-coupled receptor (GPCR) cyclic adenosine monophosphate (cAMP) cascade. GPCRs are involved in neurotransmitter action and highly expressed throughout the brain (Gerber et al. 2016). They share a seven-transmembrane-spanning α-helical segment coupled to a heterotrimeric guanine nucleotide-binding protein (G-protein). G-proteins are composed of three subunits, α, β, and γ, and classified into four distinct families depending on their Gα subunit: stimulatory G-proteins (Gα_s_, Gα_olf_), inhibitory G-proteins (Gα_i,_ Gα_o,_ Gα_t,_ Gα_z_), Gα_q_ proteins, and Gα_12/13_ proteins (Simon et al. 1991; Oldham and Hamm 2008). Binding of the respective neurotransmitters to GPCRs results in catalytic conversion of Gα-bound GDP to GTP and reduces the affinity of the Gα subunit to the Gβγ subunit complex, which subsequently dissociates. The Gα subunit then activates downstream signaling effectors. In striatal MSNs, Gα proteins target the enzyme adenylyl cyclase 5 (AC5), which is involved in generation of the second messenger cAMP. Activation of DRD1 stimulates Gα_olf_-mediated AC5 enzyme activity and increases cAMP levels, whereas activation of DRD2 leads to Gα_i_-mediated inhibition of AC5 activity and decreases cAMP levels (Stoof and Kebabian 1981; Zhuang et al. 2000; Hervé et al. 2001; Lee et al. 2002) (Fig. 2). Intracellular levels of cAMP are linked to the activity of protein kinase A (PKA), which phosphorylates downstream effector proteins including ion channels, neurotransmitter receptors, and transcription factors (Fisone et al. 2007). In striatal MSNs, an increase in cAMP and PKA leads to phosphorylation of the dopamine and cAMP-regulated phosphoprotein of 32 kDa (DARP-32) and the transcription factor cAMP-responsive element-binding protein (CREB). DARP-32 is phosphorylated at the Thr-32 residue and as such acts as an inhibitor of protein phosphatase-1 (PP-1) (Fisone et al. 2007). This in turn reduces dephosphorylation of downstream target effectors including voltage-dependent calcium channels, NMDA, AMPA, and GABA_A_ receptors, and thus has a broad impact on neuronal function (Nairn et al. 2004). The enzyme phosphodiesterase 10A (PDE10A), a dual cAMP-cGMP phosphodiesterase, constitutes another modulator of cellular cAMP and cGMP levels and is highly abundant in striatal MSNs.

#### Dysfunction of medium spiny neurons in movement disorders

It has become increasingly evident that disruption of the cAMP signaling pathway contributes to postsynaptic dysfunction that is associated with movement disorders such as dystonia, chorea, and parkinsonism (Table 1). It is hypothesized that altered dopaminergic signaling in striatal MSNs plays a key role in the pathogenesis of movement disorders. Dystonia is postulated to result from overactivity of direct pathway MSNs leading to reduced GPi activity (Fig. 1(b)). Chorea and ballism may be associated with hypofunction of indirect-pathway MSNs resulting in reduced pallidal output (Fig. 1(c)). Both mechanisms ultimately lead to inadequate GABAergic inhibition of thalamocortical projections and a hyperkinetic movement disorder. Parkinson’s disease, in contrast, is characterized by dopamine depletion in the substantia nigra leading to increased activity of striatal indirect-pathway neurons. This in turn results in enhanced inhibitory output from GPi and SNr and leads to decreased activity in thalamocortical neurons and a hypokinetic movement disorder (DeLong et al. 2007).

**Table 1:** Overview of molecular, genetic, and clinical features of postsynaptic movement disorders related to defects in the cAMP pathway in striatal medium spiny neurons

Gene	Protein	Postulated effect of mutation on protein function	Inheritance	Typical age of onset	Typical clinical features	Distinguishing clinical features	Drugs reported to have some efficacy
*ADCY5*	Adenylate cyclase 5	Gain of function	AD (familial, de novo, and somatic mosaicism)	Infancy to childhood	Generalized chorea, perioral dyskinesia, dystonia and myoclonus, lower limb spasticity, static or mildly progressive	Fluctuation in severity and frequency of MD, sleep-related episodes of hyperkinesia	Clonazepam, clobazam, acetazolamide, DBS
*PDE10A*	Phosphodiesterase 10A	Loss of function	AD (de novo) AR (biallelic, inheriting one disease allele from each parent)	Infancy to childhood	Generalized chorea, orolingual dyskinesia, static or mildly progressive	Abnormal MRI in heterozygous patients with bilateral T2-hyperintense striatal lesions	na
*GNAO1*	Gαo subunit of GPCR	Loss of function (EE phenotype) Gain of function (MD phenotype)	AD (de novo, somatic, and gonadal somaticism)	Infancy to childhood	Spectrum includes EE phenotype: neonatal-/infantile-onset seizures, infantile spasms, dyskinesia MD phenotype: Generalized chorea, facial and orolingual dyskinesia, dystonia, progressive	Severe exacerbations of chorea/ballism with autonomic dysfunction, complex motor stereotypies	Tetrabenazine and neuroleptics, topiramate, DBS especially for hyperkinetic exacerbations
*GNAL1*	Gα_olf_ subunit of GPCR	Loss of function	AD (familial, de novo) AR	Infancy (AR) Adulthood (AD)	Generalized chorea, hypertonia Progressive dystonia	na	na
*GPR88*	G-protein coupled receptor 88	Loss of function	AR	Childhood	Generalized chorea	na	na

*AD*, autosomal dominant; *AR*, autosomal recessive; *DBS*, deep brain stimulation; *EE*, epileptic encephalopathy; *MD*, movement disorder; *na*, not available

## Postsynaptic movement disorders

### Adenylate cyclase 5-related movement disorders

#### Clinical presentation

Adenylate cyclase 5 (*ADCY5*)-related disorders comprise a large phenotypic spectrum and include clinical presentations that mimic dyskinetic cerebral palsy, benign hereditary chorea, mitochondrial disorders, paroxysmal dyskinesia, myoclonus-dystonia, and recently alternating hemiplegia of childhood (Chen et al. 2014, 2015; Carapito et al. 2015; Mencacci et al. 2015; Chang et al. 2016; Westenberger et al. 2017; Douglas et al. 2017). Disease onset occurs typically in infancy or early childhood, and rarely in early adolescence (Fernandez et al. 2001). The movement disorder is hyperkinetic, mainly characterized by generalized chorea involving the limbs, face, and/or neck. The characteristic perioral and periorbital twitches, formerly described as facial myokymia, were not confirmed by EMG studies, but rather represent a mixture of myoclonic, choreic movements (Tunc et al. 2017) manifesting as orolingual dyskinesia. Limb dystonia can be a major disease feature. Additional movement abnormalities including myoclonus and lower limbs spasticity with pyramidal signs are frequently reported. Eye movement abnormalities such as saccade initiation failure and upward gaze palsy have been described in a number of patients (Chang et al. 2016). Abnormal movements may show marked fluctuation in severity and frequency and can be continuous or paroxysmal (Fernandez et al. 2001; Chen et al. 2014; Mencacci et al. 2015). The disease course is usually either static or mildly progressive over time. Many patients suffer severe and painful episodic exacerbations of the movement disorder that can last minutes to hours and may be triggered by emotional stressors, intercurrent infections, or sudden action. Sleep-related worsening of the movement disorder, in particular during drowsiness and awakening, constitutes a specific characteristic feature of *ADCY5*-related disorders. Axial hypotonia, often preceding the movement disorder, is a common finding and rarely associated with weakness (Chen et al. 2015). Cognition is usually preserved in patients or only mildly impaired. However, severely affected patients may manifest delayed motor and/or language milestones (Chen et al. 2014). Brain MR imaging is typically normal in *ADCY5*-related disorders (Chen et al. 2015).

#### Genetics

Mutations in the *ADCY5* gene were originally identified in a single five-generation German kindred with an autosomal dominant pattern of inheritance, formerly described as familial dyskinesia and facial myokymia (Fernandez et al. 2001). To date, over 80 patients from 50 affected families have been genetically confirmed (Fernandez et al. 2001; Chen et al. 2012, 2015; Carapito et al. 2015; Mencacci et al. 2015; Chang et al. 2016; Dy et al. 2016; Westenberger et al. 2017; Meijer et al. 2017; Zech et al. 2017; Douglas et al. 2017; Tunc et al. 2017; Carecchio et al. 2017). Both autosomal dominantly inherited and de novo mutations have been reported. The p.Arg418Trp variant along with the p.Arg418Gln and the p.Arg418Gly variants constitute recurrent mutations in the majority of patients and indicate a mutational hotspot at the arginine 418 residue. In vitro functional assays have demonstrated a gain of function for the p.Arg418Trp and p.Ala726Thr variants (Chen et al. 2014). Genotype-phenotype correlations suggest that the missense mutation p.Arg418Trp is associated with a more severe phenotype, while p.Arg418Gly, the p.Arg418Gln, and p.Ala726Thr show a milder phenotype (Chen et al. 2015; Chang et al. 2016). Somatic mosaicism, responsible for up to 43% of apparently de novo mutations, results in a less severe phenotype with almost complete resolution of symptoms in adulthood reported in one case (Chen et al. 2015).

#### Treatment

In *ADCY5*-related movement disorders, therapeutic trials with anticholinergics (trihexyphenidyl), dopamine antagonists (tetrabenazine), and anticonvulsants have shown limited clinical benefit. The benzodiazepines clonazepam (0.1–0.2 mg/kg) and clobazam (0.2 mg/kg) have been reported to improve sleep-related dyskinesia and myoclonic episodes (Chen et al. 2015; Chang et al. 2016). Benzodiazepines exert an indirect inhibitory effect on AC5 activity, which might counterbalance the gain of function associated with the p.Arg418Trp mutation (Dan’ura et al. 1988; Chang et al. 2016). Acetazolamide has shown a positive effect on chorea in three patients (Carecchio et al. 2017). Treatment with bilateral GPi deep brain stimulation (GPi-DBS) elicited a positive clinical response (Dy et al. 2016; Meijer et al. 2017). Two case reports showed a significant improvement of dyskinesia and dystonia after DBS (Chang et al. 2016; Meijer et al. 2017). However, the long-term efficacy of DBS in this condition is largely unknown.

#### Molecular mechanisms

The enzyme adenylyl cyclase 5, encoded by the gene *ADCY5*, constitutes the major adenylyl cyclase isoform in the brain and is enriched in the striatum, in particular the nucleus accumbens, where it accounts for 80% of AC activity (Matsuoka et al. 1997). AC5 is a membrane-bound protein that receives signals from striatal GPCRs including DRD1, DRD2, and A2A adenosine receptor (Lee et al. 2002). AC5 converts adenosine triphosphate (ATP) into cAMP upon GPCR-activation (Hanoune et al. 1997). Functional studies into *ADCY5* gain of function mutations in an in vitro HEK293 overexpression cell model demonstrated an increase in intracellular cAMP levels (Chen et al. 2014). The AC5 knockout mouse model in contrast, mimicking loss of function, exhibits a hypokinetic phenotype with parkinsonian features (Iwamoto et al. 2003). In *Adcy5*^*−*^*/*^*−*^ mice, attenuation of DRD2 signaling was associated with abnormal coordination, while attenuated locomotion activity was due to defective DRD1 signaling (Iwamoto et al. 2003). In striatal MSNs, AC5 constitutes a key enzyme involved in the modulation of dopaminergic signals and is thus tightly associated with motor control.

### Phosphodiesterase 10A-related movement disorders

#### Clinical presentation

The phenotypic spectrum of *PDE10A*-related disorders is strongly correlated to the mutation dosage. In patients carrying a single heterozygous *PDE10A* variant, disease onset occurs between 5 and 15 years of age. The movement disorder is characterized by chorea that tends to generalize over time. Esposito and colleagues recently described a patient with generalized, non-progressive chorea and diurnal fluctuation that gradually improved during the day and was absent at night (Esposito et al. 2017). The disease course is usually mildly progressive. Patients with dominant *PDE10A* mutations usually manifest normal cognition and development. Brain MR images show characteristic symmetrical bilateral T2-hyperintense lesions of the striatum (Mencacci et al. 2016; Esposito et al. 2017). In contrast, patients harboring recessive *PDE10A* mutations are more severely affected. They usually present with chorea in the first year of life. Facial involvement with orolingual dyskinesia was found in six patients of one kindred and resulted in severe dysarthria and drooling (Diggle et al. 2016). Reported patients with homozygous mutations had additional neurological features including delayed motor and speech development, cognitive decline, and axial hypotonia (Diggle et al. 2016). Focal epilepsy has been described in one patient (Diggle et al. 2016). Brain MRI of patients with recessive disease does not show any structural abnormalities of the basal ganglia, though investigation with a specific PDE10A PET ligand revealed significant loss of striatal PDE10A in one patient (Diggle et al. 2016).

#### Treatment

Management of *PDE10A*-related disorders is based on the symptomatic treatment of chorea. In other neurological disorders including Huntington’s disease (HD) and schizophrenia, PDE10A has long been considered a promising target for pharmacological treatment (Menniti et al. 2007; Raheem et al. 2016). In these disorders, perturbation of striatal output has been associated with disease pathophysiology (Raheem et al. 2016; Beaumont et al. 2016). In HD, dysfunction of indirect MSNs is thought to be responsible for the hyperkinetic movement disorder in the early stage of the disease, which is mainly characterized by chorea (Beaumont et al. 2016). Reduced levels of PDE10A have been found in HD patients and HD mouse models (Beaumont et al. 2016). Pharmacologic inhibition of PDE10A in particular enhanced activity and cortical responsiveness of indirect-pathway MSNs and restored defective basal ganglia corticostriatal circuitry, thus mimicking DRD2 agonists (Beaumont et al. 2016). Hence, PDE10A inhibitors might in the future provide a potential therapy for the hyperkinetic features of both HD disease and *PDE10A*-related disorders.

#### Genetics

To date, two recessive homozygous *PDE10A* mutations (p.Tyr107Cys and p.Ala116Pro) have been identified in eight individuals from two consanguineous families. Two recurrent de novo dominant heterozygous *PDE10A* missense mutations (p.Phe300Leu and p.Phe334Leu) have been reported in four unrelated individuals and in members of a family with an autosomal dominant mode of inheritance (Diggle et al. 2016; Mencacci et al. 2016; Esposito et al. 2017). Both recessive and dominant mutations result in loss of function and reduced levels of PDE10A in the striatum (Diggle et al. 2016; Mencacci et al. 2016). In silico modeling of the p.Phe300Leu and p.Phe334Leu variants demonstrated that the affected amino acids reside within the regulatory GAF-B-binding domain, which stimulates PDE10A activity upon binding of cAMP (Mencacci et al. 2016). In vitro studies verified severly affected cAMP-binding properties (Mencacci et al. 2016). As previously described, genotype-phenotype correlations suggest a milder phenotype associated with dominant heterozygous mutations and a more severe phenotype related to homozygous recessive mutations.

#### Molecular mechanisms

*PDE10A* encodes the enzyme phosphodiesterase 10A, a dual cAMP-cGMP phosphodiesterase, which is highly abundant in MSNs of the striatum (Coskran et al. 2006). PDE10A catalyzes the hydrolysis of cAMP and cGMP to their corresponding degradation products nucleoside 5′-monophosphate and thus regulates both cAMP and cGMP downstream signaling cascades. PDE10A is involved in the modulation of DRD1- and DRD2-activated GPCR-signaling and in the control of striatal gene expression (Strick et al. 2010; Diggle et al. 2016). Pharmacological studies revealed that inhibition of PDE10A preferentially targets indirect-pathway MSNs resulting in suppression of movement and hypokinesia (Threlfell et al. 2009). Indeed, *Pde10a*-knockout-mice and *Pde10a-*knock-in mice (p.Tyr97Cys variant) show reduced striatal PDE10A levels and manifest hypokinetic movement abnormalities (Schmidt et al. 2008; Diggle et al. 2016). In humans, biallelic mutations in the *PDE10A* gene are also associated with reduced striatal levels of PDE10A, but in contrast, a hyperkinetic movement disorder. This observation may reflect species-specific effects and is reminiscent of the situation in HD disease. In both human patients and the corresponding HD mouse models, striatal levels of PDE10A are reduced (Beaumont et al. 2016). However, HD patients typically manifest an early, hyperkinetic movement phase followed by a hypokinetic phase in the later stage of disease. However, very few HD mouse models accurately recapitulate the early hyperkinetic phase which characterizes the early stage of disease (Diggle et al. 2016). As is the case for many human movement disorders, the mouse model only partially reflects the disease evident in human patients.

### G proteinα_o_-related disorders

#### Clinical presentation

The G proteinα_o_ (GNAO1)-related phenotypic spectrum includes a spectrum of overlapping neurological phenotypes, including early-onset epileptic encephalopathy (EE), drug-resistant epilepsy with movement disorder (chorea, athetosis, dystonia, stereotypies) and movement disorder (mainly chorea and athetosis) without seizures. Patients with the epileptic encephalopathy phenotype usually manifest neonatal or infantile-onset tonic seizures or infantile spasms and exhibit distinct EEG features including burst suppression or hypsarrhythmia. Affected patients exhibit severe developmental delay and later may develop a dyskinetic movement disorder (Nakamura et al. 2013; Talvik et al. 2015; Saitsu et al. 2016; Marcé-Grau et al. 2016; Danti et al. 2017). This condition is currently classified as EEI17 (MIM no. 615473). The movement disorder phenotype is mainly characterized by progressive chorea and dystonia that usually develops in the first few years of life. Dyskinesia, in particular facial and orolingual, dystonia, and complex motor stereotypies have been commonly reported (Saitsu et al. 2016; Ananth et al. 2016; Danti et al. 2017). The onset of movement disorder is often preceded by marked hypotonia and neurodevelopmental delay. With increasing age, many patients develop severe exacerbations and suffer from episodes of refractory chorea and ballismus often accompanied by autonomic dysfunction with tachycardia, hyperthermia, hypertension, and diaphoresis (Ananth et al. 2016) (“status hyperkineticus”). Triggers often lead to these exacerbations, and may include fever, intercurrent infections, heightened emotion, and stress. Attacks often arise in clusters and can last minutes to days or even weeks (Danti et al. 2017), often requiring admission to the intensive care unit. Patients with a predominant movement disorder phenotype often show mild cognitive impairment. In patients with *GNAO1*-related disease, brain magnetic resonance imaging is usually non-specific. However, a thin abnormal corpus callosum has been commonly reported (Danti et al. 2017). Atrophy of the basal ganglia and cerebral atrophy have also been described (Ananth et al. 2016; Sakamoto et al. 2017).

#### Treatment

For *GNAO1*-related disorders, tetrabenazine, in particular in combination with neuroleptics (risperidone, haloperidol), appears to be effective for the baseline treatment of chorea (Ananth et al. 2016; Danti et al. 2017). However, clinicians should be cautious about side effects including acute dystonic reactions or malignant neuroleptic syndrome. Sakamoto reported a dramatic response to the anticonvulsant topiramate (7.5 mg/kg), an effect which might be attributed to the inhibitory action on voltage-gated Ca^2+^ channels (Sakamoto et al. 2017). Episodic exacerbations of movement disorder are often pharmacoresistant. It is of utmost importance to urgently refer these patients to the intensive care unit for dystonia management (increment of dystonia medication dosages, sedation, paralysis), adequate hydration, and continuous monitoring of cardiorespiratory functions, temperature, and laboratory parameters including creatine kinase and renal function to reduce the risk of hyperthermia, renal failure, and rhabdomyolysis. In the case of pharmaco-refractory chorea or dyskinesia, especially when it becomes life-threatening, (emergency) placement of a deep brain stimulator (DBS) into the globus pallidus internus has often resulted in an excellent clinical response (Kulkarni et al. 2016; Yilmaz et al. 2016; Danti et al. 2017).

#### Genetics

To date, *GNAO1* mutations have been identified in 43 individuals (Nakamura et al. 2013; Talvik et al. 2015; Law et al. 2015; Saitsu et al. 2016; Kulkarni et al. 2016; Marcé-Grau et al. 2016; Ananth et al. 2016; Yilmaz et al. 2016; Menke et al. 2016; Arya et al. 2017; Danti et al. 2017; Sakamoto et al. 2017; Schorling et al. 2017; Waak et al. 2017; Bruun et al. 2017). Pathogenic variants include mostly missense mutations, but also splice site mutations and one single case with a deletion (Nakamura et al. 2013; Danti et al. 2017). Mutations usually occur de novo, with somatic and gonadal mosaicism being described in several families (Nakamura et al. 2013; Yilmaz et al. 2016; Menke et al. 2016). The recurrence risk after one affected child has been estimated at 5–15% (Menke et al. 2016). In almost half of all patients, mutations arise at the highly conserved Arg209 and Glu246 residue indicating mutational hotspots. In vitro functional investigations into the molecular mechanism of 15 *GNAO1* pathogenic variants suggested genotype-phenotype correlations (Feng et al. 2017a). *GNAO1* loss of function variants was associated with epileptic encephalopathy, while gain of function variants was related to those causing predominantly movement disorders (Feng et al. 2017b). Menke and colleagues further reported that de novo missense mutations in the *GNAO1* codon 209 and 246 are predominantly associated with a movement disorder phenotype and developmental delay but without seizures (Menke et al. 2016). Based on a review of literature, Schorling et al. described a female preponderance for the EE phenotype, suggesting that predilection for epilepsy might be a gender-specific effect in *GNAO1*-related disorders (Schorling et al. 2017). The movement disorder phenotype appears to affect both sexes equally.

#### Molecular mechanisms

*GNAO1* encodes the alpha-o subunit (Gα_o_) of G-proteins. G_o_ are the most abundant G-proteins in brain tissue, particularly in neuronal synapses (Jiang and Bajpayee 2009). They regulate multiple intracellular effectors and associated signaling cascades including ion channels, enzymes, and small GTPases (Jiang and Bajpayee 2009). At the presynaptic level, G_o_ proteins further mediate autoinhibitory effects of several neurotransmitters on their receptors (Brown and Sihra 2008). Gα_o_ subunits are specifically involved in the inhibition of voltage-gate Ca^2+^ channels and activation of inwardly rectifying K^+^ channels (Simon et al. 1991; Schorling et al. 2017). Knockdown of Gα_o_ proteins in mice (α_o_−/−) results in hyperactive behavior and motor abnormalities including generalized tremor and impaired motor control, as well as occasional seizures, hyperalgesia, and shortened lifespan (Jiang et al. 1998). A knock-in mutant mouse model (*Gnao1*^*+/G184S*^) exhibits a severe seizure phenotype and premature death (Kehrl et al. 2014). The mutant mice exhibit elevated frequency of interictal epileptiform discharges on EEG but no overt brain morphology changes were seen.

### G proteinα_olf_-related dystonia

#### Clinical presentation

G proteinα_olf_ (*GNAL1*)-related disorders were first reported in 2012, in adult-onset primary torsion dystonia (DYT 25, primary torsion dystonia) (Bressman et al. 1994; Fuchs et al. 2012). Disease onset occurs in the third or fourth decade of life. Dystonia is usually initially focal and affects predominantly the craniocervical region in most patients. With ongoing disease, dystonia progresses and typically leads to more extensive cervical or laryngeal involvement and less commonly truncal or limb involvement. Recently, Masuho et al. identified two affected individuals in a large consanguineous kindred who presented with childhood-onset dystonia (Masuho et al. 2016). Both siblings presented with hypertonia at the age of 1 year and developed generalized dystonia over time. Initial motor and language development was normal.

#### Treatment

In *GNAL1*-associated dystonia, a therapeutic trial with levodopa was not beneficial (Bressman et al. 1994). Data on treatment with other anti-dystonic agents is scarce to date.

#### Genetics

*GNAL1* mutations are inherited in an autosomal dominant manner with reduced penetrance (Carecchio et al. 2016). De novo heterozygous *GNAL1* mutations have also been described in three patients with seemingly sporadic dystonia and negative family history (Dobričić et al. 2014; Ziegan et al. 2014). Recently, autosomal recessive homozygous missense mutations in the *GNAL1* gene have been identified in a consanguineous kindred with childhood-onset dystonia (Masuho et al. 2016). In vitro functional assays have demonstrated attenuated DRD1 response for the nonsense mutant p.Ser293* and impaired association of the Gα_olf_ subunit with the corresponding Gβγ subunit for the missense mutant p.Val137Met, thereby indicating loss of function.

#### Molecular mechanisms

*GNAL1* encodes the stimulatory G-protein alpha subunit Gα_olf_. Gα_olf_ belong to the stimulating G-proteins and couple “direct pathway” DRD1 and “indirect-pathway” A2 adenosine receptors to the activation of AC5 (Corvol et al. 2001; Vemula et al. 2013). Gα_olf_ are enriched in striosomes, which are clusters of striatal MSNs that project to the SNpc (Crittenden and Graybiel 2011). An imbalance of the striatal striosome activity in relation to the surrounding matrix has been postulated to contribute to the development of hyperkinetic movement disorders (Fuchs et al. 2012). A *Gnal*^*+*^*/*^*−*^ knockout mouse model has been used to study L-DOPA-induced dyskinesia in parkinsonism (Alcacer et al. 2012). In the dopamine-denervated striatum, L-DOPA induces DRD1 signaling through the cAMP pathway including PKA and DARP-32. Striatonigral lesions of *Gnal*^*+*^*/*^*−*^ mice lead to upregulation of Gα_olf_ and induce dyskinesia upon chronic treatment with L-DOPA.

### GPR88-related chorea

#### Clinical presentation and genetics

The phenotypic spectrum of *GPR88*-related movement disorder so far includes only four individuals from one consanguineous kindred (Alkufri et al. 2016). The female siblings presented with speech delay and learning disability and developed chorea at the age of 8–9 years. The movement disorder affected mainly the face and hands, but choreiform movements were also noted in the shoulders, pelvis, and thighs. Alkufri et al. identified a homozygous nonsense mutation in *GPR88 gene* encoding an orphan G-protein-coupled receptor (Alkufri et al. 2016).

#### Molecular mechanisms

GPR88 is highly expressed in both DRD1- and DRD2-expressing MSNs of the striatum (Massart et al. 2009; Quintana et al. 2012). GPR88 deficiency in a knockout mouse model (*Gpr88*^*Cre*^*/*^*Cre*^) leads to enhanced excitability of DRD1- and DRD2-expressing striatal MSNs owing to increased glutamate receptor phosphorylation and altered GABA_A_ receptor composition (Quintana et al. 2012). The *Gpr88*^*Cre*^*/*^*Cre*^ mice show increased locomotion, hyperactivity in novel environment, and stereotypic behavior abnormalities reminiscent of striatal dysfunction (Meirsman et al. 2016).

## Other genetic movement disorders associated with secondary postsynaptic dysfunction

### DYT1 early-onset dystonia

#### Clinical presentation and genetics

DYT1 dystonia is a hereditary early-onset movement disorder caused by mutations in *TOR1A* encoding the protein torsin A. Patients manifest with isolated dystonia in childhood or adolescence, usually without any other associated neurological abnormalities (Ozelius and Lubarr 1993). Though not part of the initial presentation, executive dysfunction and psychiatric comorbidities such as mood and anxiety disorders have been described in DYT1 dystonia (Jahanshahi 2017). In the early course of disease, dystonia usually affects one (usually lower) limb and is often related to specific actions (action-induced or task-specific dystonia). Over time, dystonia usually progresses and becomes segmental, multifocal, or generalized in 60–70% of all patients (Ozelius and Lubarr 1993). DYT1 dystonia shows an autosomal dominant mode of inheritance and manifests with reduced penetrance, estimated at 30%. The majority of patients harbor a three base pair deletion c.907_909delGAG deletion, though three additional in-frame deletions have been reported singly in other individuals (Ozelius and Lubarr 1993).

#### Molecular mechanisms

Although the exact function of torsin A is yet to be fully elucidated, it is thought to shuttle between the endoplasmic reticulum (ER) and the nuclear envelope (NE) for several physiological functions including ER-associated degradation, dopamine release and metabolism, synaptic shuttling of mRNAs, and cytoskeleton dynamics (Ozelius and Lubarr 1993). Several studies have investigated the role of torsin A in dopamine neurotransmission in striatal neurons. Data from three different DYT transgenic mouse models suggest a role for presynaptic dysfunction in dopaminergic neurons owing to impaired dopamine release (Bao et al. 2010; Page et al. 2010). However, electrophysiological studies in striatal slice cultures from a transgenic DYT1 mouse model also revealed postsynaptic alterations. Activation of postsynaptic DRD2 resulted in a paradoxical excitatory effect in striatal cholinergic interneurons leading to inappropriate firing activity (Pisani et al. 2006). MSNs of transgenic mice showed decreased surface expression of postsynaptic DRD2 with deficient G-protein coupling (Napolitano et al. 2010). Further studies investigated a potential DRD2 trafficking defect due to reduced torsin A chaperone activity. This hypothesis was corroborated by data demonstrating a direct interaction between torsin A and DRD2 and PET imaging studies demonstrating decreased DRD2 availability in brains of DYT1 patients (Torres et al. 2004; Carbon et al. 2009).

### Monogenic forms of parkinsonism/Parkinson’s disease

#### Clinical presentation and genetics

Parkinson’s disease (PD) represents the second most common neurodegenerative disorder in adults and most commonly occurs sporadically (Kalia and Lang 2015). However, approximately 5–10% of patients have a monogenic form of the disease with an either autosomal recessive or dominant mode of inheritance (Lin and Farrer 2014). In these monogenic forms, disease onset typically occurs in childhood (juvenile onset parkinsonism, usually < 20 years) or adulthood before the age of 40–45 (early-onset parkinsonism) (Puschmann 2013; Bonifati 2014). PD is neuropathologically characterized by progressive loss of nigrostriatal dopaminergic neurons leading to the typical clinical triad of bradykinesia/akinesia, rigidity, and tremor. In the monogenic early-onset forms of PD, additional neurological features including neurodevelopmental delay, intellectual disability, psychiatric comorbidities, and epilepsy are commonly reported. To date, several genes have been associated with juvenile, atypical parkinsonism (*ATP13A2*, *PLA2G6*, *FBX07*, *DNAJC6*, *SYNJ1*) and early-onset parkinsonism (*SNCA*, *PARK2*, *PINK1*, *DJ1*) (Bonifati 2014).

#### Molecular mechanisms

Genes associated with early-onset parkinsonism are mainly involved in disruption of presynaptic function (Bonifati 2014). Pathogenic variants have been shown to impair protein trafficking, autophagy, and mitochondrial function culminating in loss of dopaminergic neurons (Lynch-Day et al. 2012; Pickrell and Youle 2015; Hunn et al. 2015). Many of the affected proteins in PD may also have other effects in different synaptic compartments, which remain yet to be fully elucidated. Indeed, in early-onset PD, there is emerging evidence for postsynaptic alterations that may contribute to the disease pathology. For example, Parkin, encoded by the gene *PARK2*, has been shown to localize to not only presynaptic but also postsynaptic terminals (Sassone et al. 2017). At the postsynaptic terminal, Parkin colocalizes with the postsynaptic density marker PSD-95. Through interaction with PSD-95, Parkin is suggested to regulate trafficking, anchoring, and clustering of membrane surface receptors (Sassone et al. 2017). Parkin is further involved in the mono-ubiquitination of PICK1, a synaptic scaffold protein that regulates the trafficking of several neurotransmitter receptors, ion channels, and enzymes (Joch et al. 2007). Further studies demonstrated that Parkin modulates postsynaptic glutamate receptors. Loss of Parkin leads to an increase in excitatory activity, which ultimately results in exitotoxic dopaminergic cell death (Sassone et al. 2017). Further studies are warranted to elucidate postsynaptic disease mechanisms in genetic early-onset PD. Overall, investigation of postsynaptic alterations in monogenic PD may provide insights into more common forms of PD.

## Conclusion

Over the past few years, a number of genetic movement disorders have been identified where defects in postsynaptic MSN function are thought to play a crucial role in disease pathogenesis. Mutations in genes such as *ADCY5*, *PDE10A*, *GNAO1*, *GNAL1*, and *GPR88* affect key proteins of the postsynaptic cAMP signaling pathway, which mediate the effects of dopaminergic neurotransmission in striatal MSNs. On a molecular level, loss or gain of function pathogenic variants differentially impact on the signaling cascade but result in hypo- or hyperfunctional dopaminergic signaling in striatal MSNs.

From a clinical viewpoint, these genetic diseases which align to a common disease pathway also manifest a number of overlapping clinical features. All are characterized by prominent, early-onset movement disorders with hyperkinetic manifestations such as chorea and dyskinesia. Facial involvement is commonly reported in *ADCY5-*, *PDE10A-*, *GNAO1-*, and *GPR88*-related disorders. Despite these similarities, the course of disease and specific distinct phenotypic features may help to discriminate them clinically. Indeed, *ADCY5-* and *PDE10A*-related disorders seem to show a static or mildly progressive course, while *GNAO1*-related movement disorders are characterized by progressive chorea which can become life-threatening in some patients. Distinguishing clinical features may further include sleep-related phenomena and marked fluctuation in *ADCY5* disease, abnormal MRI features in dominant *PDE10A* disease, and severe exacerbations associated with autonomic dysfunction in patients with *GNAO1* mutations.

Given these substantially overlapping phenotypes, establishing a definitive diagnosis is often not straightforward. Furthermore, with increasing patient diagnoses, the molecular and clinical spectrum is likely to further expand, with the identification of atypical disease phenotypes. Implementation of next-generation sequencing techniques in clinics has already translated into better diagnostics of these rare postsynaptic disorders. For many of these disorders, a diagnostic whole-exome approach or multiple-gene panel testing may be the most efficient method of reaching a confirmatory diagnosis. Despite these genetic advances, clinicians still face the enormous unmet need for disease-specific personalized therapies, as many of these disorders are pharmacoresistant and challenging to treat with conventional, currently available drugs. Precision medicine approaches, targeting the specific gene defect may provide a better long-term strategy to overcome this gap. Gene therapy and RNA manipulation techniques represent attractive new technologies to approach a patient’s specific genetic condition. Future identification of specific therapies targeting the cAMP pathway, a critical cellular signaling pathway in striatal MSNs, may revolutionize the treatment of these severe genetic movement disorders.
